# Efficacy and safety of a natural mineral water rich in magnesium and sulphate for bowel function: a double-blind, randomized, placebo-controlled study

**DOI:** 10.1007/s00394-015-1094-8

**Published:** 2015-11-18

**Authors:** Gordana Bothe, Aljaz Coh, Annegret Auinger

**Affiliations:** 1 Analyze & Realize GmbH, Waldseeweg 6, 13467 Berlin, Germany; 2 Droga Kolinska, d.d., Kolinska ulica 1, 1544 Ljubljana, Slovenia

**Keywords:** Bowel function, Stool frequency and consistency, Natural mineral water, Clinical trial

## Abstract

**Purpose:**

The present placebo-controlled, double-blind, randomized trial aimed to investigate whether a natural mineral water rich in magnesium sulphate and sodium sulphate (Donat Mg) may help to improve bowel function.

**Methods:**

A total of 106 otherwise healthy subjects with functional constipation were randomly assigned to consume 300 or 500 mL of a natural mineral water as compared to placebo water, over a course of 6 weeks. The 300-mL arms were terminated due to the results of a planned interim analysis. Subjects documented the complete spontaneous bowel movements, spontaneous and overall bowel movements/week, stool consistency, gastrointestinal symptoms and general well-being in a diary. Change in the number of complete spontaneous bowel movements was defined as the primary outcome.

**Results:**

For the 75 subjects in the 500-mL arms, the change in the number of complete spontaneous bowel movements per week tended to be higher in the active group when compared to placebo after 6 weeks (*T*2 = 1.8; *p*_value_ = 0.036; one-sided). The mean number of spontaneous bowel movements significantly increased over the course of the study, with significant differences between study arms considering the whole study time (*F* test = 4.743; *p*_time × group_ = 0.010, 2-sided). Stool consistency of spontaneous bowel movements (*p* < 0.001) and the subjectively perceived symptoms concerning constipation (*p* = 0.005) improved significantly with the natural mineral water as compared to placebo.

**Conclusions:**

The daily consumption of a natural mineral water rich in magnesium sulphate and sodium sulphate improved bowel movement frequency and stool consistency in subjects with functional constipation. Moreover, the subjects’ health-related quality of life improved.

**Clinical Trial Registration:**

EudraCT No 2012-005130-11.

## Introduction

Constipation is one of the leading bowel conditions affecting health-related quality of life but also comprising other complaints resulting in substantial healthcare costs [1–3]. Functional constipation is a functional gastrointestinal disorder characterized by straining during defecation, lumpy or hard stool, sensation of incomplete evacuation and infrequent bowel movements without evident organic or structural diseases [4]. An integrative review of eleven studies conducted in Asian, South American and European countries revealed a prevalence of constipation ranging from 2.6 to 26.9 % in the general adult population [5]. In Europe, a mean prevalence rate of 17.1 % was reported [6].

In principle, the spectrum of stool types in the general healthy population is wide including hard, fragmented lumps to sausage-like or snake-like forms up to mushy material, which correlates with transit time and faecal bulk [7]. The water content of stool usually ranges from 70 to 80 %, while constipated subjects have a harder stool with water content below 70 % [8]. Stool consistency is frequently related to bowel movement, which also differs considerably between individuals, with a mean frequency of one stool per day reported for people living in Western countries [9].

Generally, the aetiology of constipation is very complex and mainly influenced by dietary factors including drinking habits and a sedentary lifestyle; hence, changes in lifestyle and diet are usually the first recommendation for constipated subjects [10]. The use of laxatives, bulking agents, stool softeners or other remedies is recommended if lifestyle modifications fail. These might, however, be associated with side effects; furthermore, according to a recent survey in ten European countries, 28 % of subjects were dissatisfied with their current constipation treatment [10].

In a cross-sectional study on Japanese women, a low intake of magnesium and water from food was inversely associated with the prevalence of functional constipation. Indeed, magnesium salts such as magnesium sulphate are known for their osmotic effects accelerating intestinal transit time and leading to better stool consistency [11, 12]. Natural mineral waters rich in magnesium salts are therefore thought to improve bowel function. So far, controlled clinical trials assessing the effect of mineral-rich water on bowel function have not been frequently reported [13–15]. The aim of the present randomized, controlled interventional study was to examine the effect of a natural mineral water rich in magnesium sulphate and sodium sulphate as compared to placebo on bowel function in otherwise healthy adults with functional constipation.

## Methods

### Trial design

The study was designed as a mono-centre, parallel-treatment, multi-dose, double-blind study in subjects with functional constipation. The trial incorporated a planned, adaptive interim analysis to adjust for sample size and to evaluate the different doses with regard to the main study objective. The first part of the study was performed with four study arms: two arms with a daily dose of 300 mL (active and placebo) and two arms with a daily dose of 500 mL (active and placebo). Following the interim analysis, the lower-volume/lower-dose arms were terminated, and the final sample size (for the 500 mL dose) was adjusted. Results for the lower dose are not presented.

The study was approved by the Ethics Committee (Office for Health and Social Affairs, Berlin, Germany) and by the Competent Authority (Federal Institute for Drugs and Medical Devices, Bonn, Germany). It was conducted in compliance with the Declaration of Helsinki as well as the German Pharmaceuticals Act, the principles of ICH-GCP, and the German GCP-V. The study was registered in the European Clinical Trials Database (EudraCT) as EudraCT No 2012-005130-11. Participants gave written informed consent prior to the study.

### Subjects

A total of 106 otherwise healthy subjects with functional constipation were enrolled to the study site in Berlin, Germany. Subjects aged 18–70 with functional constipation according to ROME III criteria having two-to-four bowel movements per week during the preceding months were included [4, 16]. They were asked to adhere to their former diet and physical activity and had to be used to consuming at least 300 mL of water (incl. tea). Women of childbearing potential had to agree to use contraception methods. The exclusion criteria were as follows: acute gastritis and enteritis, bleeding tendency and risk of rupture in the intestinal tract, disorders in motility and secretion in the digestive tract, acute or chronic disease of the gastrointestinal tract, irritable bowel syndrome, abdominal pain, abdominal surgery within the last 6 months prior to study start, known pelvic floor dysfunction, susceptibility to development of kidney stones, hyperresorptive hypercalciuria with urinary stones, urinary infections with *E. coli*, renal insufficiency, acute or chronic kidney or urinary tract disease, alkalosis, severe respiratory disease, cardiovascular system insufficiency, acute inflammatory diseases, dehydrated conditions, restricted fluid tolerance, acute or chronic neurological or psychiatric illness, weight loss of ≥3 kg within the last 3 months prior to the study, clinically relevant excursions of laboratory parameters, BMI > 35 kg/m2, thyroid dysfunction, known sensitivity to the ingredients of the product, use of any preparations that could affect the gastrointestinal tract during the last 2 weeks and during the study (except rescue medication, a bisacodyl suppository that could be used in case of no bowel movements for 4 days; max. four suppositories were permitted during the entire study), use of sympathomimetics and cardiac glycosides, supplementation of magnesium, vitamins or other minerals during the study, intake of mineral water other than the study product during the study, pregnancy or nursing, drug, alcohol or medication abuse, participation in another clinical trial during the last 30 days prior to study start, relationship with or dependence on the sponsor or the investigator, problems with complying with or following the protocol due to language difficulties, or commitment to an institution by virtue of an order issued either by the judicial or the administrative authorities.

Furthermore, subjects had to fulfil the following randomisation criteria during the run-in period before obtaining the investigational product (assessed per subject diary): two-to-four bowel movements per week and intake of at least 300 mL water (incl. tea) daily.

### Intervention

During the study period of 6 weeks, the subjects consumed their daily dose of Donat Mg natural mineral water or placebo in two portions: prior to breakfast and in the evening before dinner. Donat Mg natural mineral water is derived from a spring in Rogaska Slatina, Slovenia. It is enriched by minerals from dissolving rocks 280-600 m under ground providing Donat Mg with 13 g/L of dissolved mineral substances. Both the mineral water and the placebo were produced and bottled by Droga Kolinska, d.d (Slovenia). Main ingredients of Donat Mg natural mineral water are sodium (1600 mg/L), magnesium (1000 mg/L), calcium (370 mg/L), sulphate (2000 mg/L), and hydrogen carbonate (7600 mg/L). The placebo was sparkling water derived from another spring in Rogaska Slatina with a low content of minerals (<1 mg/L sodium, 30 mg/L magnesium, 73 mg/L calcium, 17 mg/L sulphate, 390 mg/L hydrogen carbonate) and a quantity of CO_2_ (3.5 g/L) comparable to the active product.

The clinical phase of the study included a run-in period of 10 ± 2 days and an intervention period of 6 weeks ± 3 days. A total of five visits were performed: a screening visit, a baseline visit (after the run-in period), a telephone visit 7 ± 3 days after baseline, a control visit 21 ± 3 days after baseline and the final visit 42 ± 3 days after baseline.

Compliance was checked by counting returned unused investigational product and assessing the trial duration. The accepted compliance rate was defined as 75–125 % of the correct quantity of investigational product and max. 3 days deviation from the 6-week study period.

### Outcome measures

The primary outcome was the subject-rated change in the number of complete spontaneous bowel movements (CSBMs) per week between placebo and active over the course of the study. A CSBM was defined as a bowel movement with sensation of complete evacuation and with no laxative/enema in the 24 h preceding the bowel movement. In addition, the number of overall bowel movements (BM), the number of spontaneous bowel movements (SBM, bowel movements with no laxative/enema in the 24 h preceding the bowel movement) and the number of complete bowel movements (CBM, defined as bowel movements with a sensation of complete evacuation) were assessed. Subjects were asked to record their stool consistency using Bristol Stool Form Scale (BSFS) questionnaire [17].

Subjects had to document their daily bowel movements, the respective stool form, the sensation of complete evacuation following defecation (yes/no) and whether they had used any rescue medication in a daily diary. In addition, subjects had to fill in the questionnaires Gastrointestinal Symptoms Rating Scale (GSRS) [18], the Short Form 12 Health Survey Questionnaire (SF-12) [19] and the short form of the international physical activity questionnaire (IPAQ-SF) at each visit post screening [20]. Eating and drinking habits were recorded in a diary on 3 days of each week (to calculate the mean answer to the items regarding intake of certain foods/day, the following score was used: none = 1, once = 2, twice or three times = 3, more than three times = 4; the intake of liquids was recorded in mL). The efficacy of the investigational products was evaluated by the participants and the investigator independently at the end of the study by means of a global-scaled evaluation with “very good”, “good”, “moderate” or “poor”.

Biochemical parameters such as liver function and lipid parameters as well as blood pressure and heart rate were assessed at screening and at the end of the intervention.

### Adaptive design and statistics

The adaptive interim analysis was scheduled to take place when data from 50 % of originally planned number of subjects were available [21]. The analysis was done by an independent statistician using the full analysis set (FAS). An Independent Data Monitoring Committee (IDMC) was in place to provide appropriate recommendations to the trial sponsor while all staff involved in the conduct of the trial remained blind to the results of the interim analysis. Two sets of one-sided confirmatory hypotheses were planned to be tested at the interim and final analysis for the primary efficacy variable dCSBM in each of the dose groups (i.e. 300 mL each verum or placebo; 500 mL each verum or placebo) (Table 1).

**Table 1:** Splitting of the overall type I error

Alpha (one-sided)	Stage	Analysis	Critical value to reject H0			
			H(01)		H(02)	
			Test statistic	Alpha (one-sided)	Test statistic	Alpha (one-sided)
0.025	1	Interim	3.011	0.0013	2.797	0.0026
	2	Final	2.257	0.0120	1.977	0.0240

The trial results were analysed according to a two-stage adaptive group sequential design with one interim analysis using O’Brien and Fleming stopping boundaries [21]. Each hypothesis was tested (at the interim and final analysis) with the nonparametric one-sided Mann–Whitney *U* test. The inverse-normal method was used to combine the *p* values for each of the two sets of one-sided confirmatory hypotheses [22].

The overall experiment-wise significance level [i.e. the family-wise error rate (FWER)] was set to = 0.025 (one-sided). Adjustment of the significance level was performed to control the overall type I error = 0.025 (one-sided). In the interim analysis, the null hypotheses could not be rejected for any of the dose levels. The IDMC recommended stopping the low-volume-dose groups and to adjust the sample size for the higher dose. The final analysis of the primary endpoint was only performed for the 500-mL dose with the test statistic *T*2 critical value to reject the null hypothesis of 1.977. The secondary outcome parameters were tested in the exploratory sense using the nonparametric procedures Mann–Whitney *U* test, Wilcoxon test and Chi-square test. Changes in clinical parameters over time were analysed by using exploratory ANOVA. The analysis was performed using the FAS and at least for the primary outcome in addition using the valid case analysis set (VCAS).

### Sample size estimation

For the sample size estimation before the interim analysis, the calculation was based on the assumption of an effect size of 0.7 for the lower dose and 1.0 for the higher dose. The sample size re-estimation for the study continuation with the higher dose was calculated based on the target conditional power using the procedure suggested by Chang [23]. The initial estimates and the observed treatment difference and standard deviation at the interim analysis were considered. The conditional power was set to 80 %. Critical value α2 for the final analysis was set to 0.0240.

## Results

### Subject recruitment

**Fig. 1:**
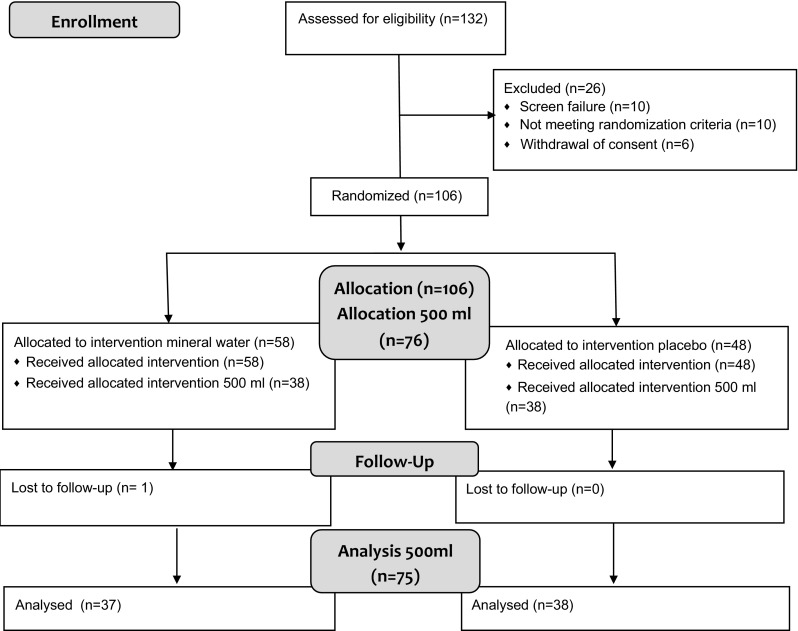
Subject flow chart

Out of the 132 subjects assessed for eligibility, 106 were randomized (Fig. 1); 30 subjects thereof were allocated to the 300-mL arms, which were terminated after the interim analysis (data not shown). Of the 76 subjects allocated to the 500-mL arms, 75 were included in the FAS population (one subject had no data except baseline).

### Characteristics of study subjects at baseline

Of the 75 subjects in the 500-mL arms, 63 were female and 12 were male with functional constipation according to ROME III (Table 2). The baseline characteristics, including the eating and drinking habits and physical activity did not differ between interventional groups.

**Table 2:** Baseline characteristics of study subjects

	Total	Active	Placebo	*p*
Gender (f/m)	63/12	31/6	32/6	0.960
Age	46.4 ± 12.6	46.8 ± 12.9	46.0 ± 12.5	0.707
Weight (cm)	70.6 ± 11.9	71.1 ± 13.6	70.2 ± 10.2	0.899
Systolic blood pressure (mmHg)	115.2 ± 12.1	113.9 ± 11.3	116.5 ± 12.9	0.332
Diastolic blood pressure (mmHg)	75.4 ± 6.6	74.9 ± 6.2	75.9 ± 7.0	0.419

*p* values (Mann–Whitney *U* test) for differences between study arms

### Bowel movements

All bowel movements analysed were spontaneous; hence, the results given for CSBMs are also valid for CBM assessment and those for SBMs accordingly for BM assessment. The change in the number of complete spontaneous bowel movements per week tended to be higher in the active group as compared to placebo after 6 weeks (test statistic *T*2 = 1.8; combined *p*_value_ = 0.036 with a confirmatory significance level of 0.024; one-sided). There was a trend between the active group and the placebo group regarding the changes in CSBMs over time (*F* test: 2.992; *p*_time × group_ = 0.054; Table 3).

**Table 3:** Mean (SD) number of CSBMs per week and SBMs/BMs per week during a 6-week intervention with mineral-rich water or placebo water

	Active		Placebo		*p*_U_
	Mean	SD	Mean	SD	
CSBMs per week					
Baseline	0.68	(1.11)	0.79	(1.10)	0.629
Week 3	2.19	(3.19)	1.26	(1.64)	0.353
Week 6	2.14	(2.67)	1.16	(1.50)	0.173
*p*_ANOVA_	0.054				
SBMs/BMs per week					
Baseline	3.38	(1.26)	3.03	(0.92)	0.329
Week 3	6.14	(3.32)	4.45	(2.09)	0.006
Week 6	6.62	(3.20)	4.47	(2.20)	0.001
*p*_ANOVA_	0.010				

*p*_U_ values (Mann–Whitney *U* test) for differences between study arms

*p*_ANOVA_ for RM-ANOVA (time × group interaction)

The mean number of SBMs/BMs almost doubled after 6 weeks of drinking the mineral-rich water, with significant differences between study arms considering the whole study time (*F* test = 4.743; *p*_time × group_ = 0.010, Table 3). After three and 6 weeks, respectively, the number in SBMs/BMs was significantly higher in subjects drinking the mineral-rich water as compared to those drinking placebo water (*p* = 0.006 and *p* = 0.001, respectively).

Similar results were shown for VCAS; there were no statistically significant differences between the active and placebo groups in changes in CSBMs per week (*p* = 0.154), while for the SBMs/BMs the groups differed significantly (*p* = 0.024).

### Stool consistency

**Fig. 2:**
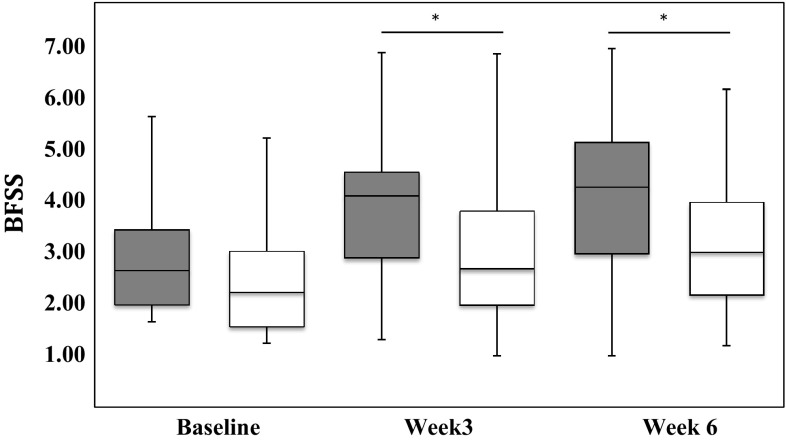
Stool consistency SBM/BM using bristol stool form scale (BSFS type, median, interquartile range, range) over the course of the intervention (*grey* active group, *white* placebo group). Bristol stool score—1 for hard lumps to seven watery stools. *p values (Mann–Whitney *U* test) for differences between study arms

With regards to the consistency of SBMs/BMs, 78.4 % in the active group and 60.5 % in the placebo group reported that the stool became softer after 6 weeks of intake, while 8.1 % and 23.7 % in the corresponding groups showed a harder stool as compared to baseline (*p*_active_ < 0.001; *p*_placebo_ = 0.012). At week 3 and 6, subjects in the active group had a significant softer stool than the placebo group (*p* = 0.001 and *p* < 0.001, respectively; Fig. 2). The stool consistency of SBMs/BMs throughout the study differed significantly between study groups (*F* test = 12.376; *p*_time × group_ < 0.001).

### Gastrointestinal symptoms and general well-being

The gastrointestinal symptoms were assessed by means of the GSRS. In line with the increased number of bowel movements and stool consistency score, subjects drinking the mineral-rich water experienced an improvement in the symptoms related to constipation throughout the study (*p* = 0.005), while other symptom clusters and the total sum score did not differ between study arms (Table 4).

**Table 4:** Mean (SD) of gastrointestinal symptom rating scale (GSRS)

	Active		Placebo		*p*
	Mean	SD	Mean	SD	
GSRS (reflux)					
Baseline	1.54	0.96	1.37	0.72	0.413
Week 6	1.39	0.63	1.45	0.79	0.990
*p*_UΔ_	0.348				
GSRS (abdominal pain)					
Baseline	1.90	0.74	1.96	0.80	0.797
Week 6	1.66	0.74	1.68	0.72	0.773
*p*_UΔ_	0.944				
GSRS (indigestion)					
Baseline	2.75	1.19	2.72	1.02	0.924
Week 6	2.26	1.13	2.30	0.99	0.617
*p*_UΔ_	0.915				
GSRS (diarrhoea)					
Baseline	1.23	0.49	1.23	0.56	0.492
Week 6	1.64	1.04	1.35	0.55	0.392
*p*_UΔ_	0.335				
GSRS (constipation)					
Baseline	3.82	1.48	3.59	1.38	0.419
Week 6	1.81	0.79	2.56	1.14	0.001
*p*_UΔ_	0.005				
GSRS (total)					
Baseline	2.33	0.76	2.26	0.70	0.722
Week 6	1.81	0.58	1.92	0.66	0.442
*p*_UΔ_	0.327				

*p*_UΔ_ values (Mann–Whitney *U* test) for differences between study arms in changes in GSRS dimension between baseline and final visit

*p* values (Mann–Whitney *U* test) for differences between study arms

In addition, there were significant differences between the mineral-rich water group and the placebo group with regards to changes in the SF-12 sum score from baseline to week 6 (*p* = 0.017).

### Physical performance and dietary habits

Physical activity was not changed in any of the study groups over the course of the study (*p* = 0.628). There was no significant difference between study groups in the consumption of beverages throughout the entire study. At the end of the intervention, the active group ate slightly more dried fruits (from 1.04 at baseline to 1.15 at 6 weeks) and carrots/potatoes (from 1.53 at baseline to 1.68 at 6 weeks) as compared to placebo (1.04 at baseline to 1.03 at 6 weeks for dried fruits, *p* = 0.04; 1.68 at baseline to 1.60 at 6 weeks for carrots/potatoes; *p* = 0.03).

### Global evaluation of the efficacy

At the end of the study, 94.5 % of subjects in the mineral-rich water group—compared to 57.9 % in the placebo group—rated the global evaluation of efficacy as “very good” or “good”. Similarly, the physicians rated the mineral-rich water as “very good” or “good” for 97.2 % of the participants, while they gave the same rating for 57.9 % of the participants consuming the placebo water. Both participants and physicians rated the efficacy of the mineral-rich water better than the placebo water (*p*_chi_ = 0.001 and *p*_chi_ < 0.001, respectively).

### Safety evaluation, laboratory parameters and use of rescue medication

A total of 16 adverse events occurred during the study period in the 500-mL arm with no statistical difference between the mineral water group and placebo group (*p* = 0.571). Twelve occurred in the active group, and another four events were recorded in the placebo group. One case in the active group might be related to the intervention. In two cases of the active group, the causality of the adverse event was probably related to the intervention. One adverse event in the active group was classified as serious but was not related to the intervention.

None of the measured clinical parameters including liver enzymes, triglycerides, cholesterol, HDL-C, LDL-C, blood pressure and heart rate values differed between study groups.

With regards to rescue medication, no difference was observed between groups regarding the intake of rescue medication (*p* = 0.240).

There were no statistically significant differences between groups in deviation of the actual intake of investigational products from expected intake (*p* > 0.1). The compliance rate considering the intake of the investigational product was 102 % ± 8.6 in the active group and 105 % ± 9.4 in the placebo group.

## Discussion

The present placebo-controlled, randomized, double-blind intervention study in subjects with functional constipation provides clinical evidence that drinking a mineral water rich in magnesium sulphate and sodium sulphate over a course of 6 weeks can confer significant benefits for digestive health. The improvement in the constipation symptoms as reflected by an increased number of CSBMs and overall bowel movements per week and a softer stool in comparison with a water low in minerals confirmed previous assumptions of the digestion stimulating effects of waters naturally rich in minerals. Indeed, the number of CSBMs/week more than tripled within the course of the study in subjects drinking the mineral-rich water, which is comparable to the effect of psyllium and dried plums demonstrating an increase in the mean number of CSBMs/week by more than a double [24].

In line with the improved objective parameters of bowel function, study participants experienced an improvement in the GSRS dimension constipation exceeding the minimal clinically relevant score change of 0.5 [25] together with an overall increase in the health-related quality of life.

The change in the number of CSBMs per week was chosen as the primary endpoint as it is considered the more sensitive assessment with regards to the overall bowel function as information on the completeness of bowel movement is also provided [26]. Since there was no adequate and well-controlled trial with the investigational product available at the time the current study was planned, an adaptive study design was applied to adjust the sample size, if necessary or to terminate the entire trial, the low- (300-mL) or the high- (500-mL) volume dose arms earlier if efficacy in the primary endpoint is lacking or already achieved at the interim analysis. In terms of CSBMs, participants did not seem to benefit from drinking 300 mL of the mineral water per day, and therefore these arms of the study were closed, while the sample size of the 500-mL dose group was increased based on the results of the interim analysis. Given the outcomes of this clinical trial, the intake of 500 mL of Donat Mg per day seems to improve CSBMs but also overall bowel movements and consistency more efficiently than the 300-mL dose.

As dietary habits and lifestyle factors are thought to impact bowel function, the subjects’ eating and drinking habits and physical activity levels were assessed. Only a marginal increase in the consumption of dried fruits was observed in the active group as compared to placebo over the course of the study. Given the semi-quantitative nature of the dietary assessment and the observed small differences between study groups, it is highly unlikely that this change might have had an impact on the study results.

The outcome of the present clinical trial is in agreement with another recently published study comparing different dosages of a mineral water with placebo. In that trial, the daily consumption of 1 L magnesium sulphate-rich mineral water (119 mg/L magnesium, 1530 mg/L sulphate) reduced constipation and improved stool consistency in functionally constipated women [13]. Although the study group was limited to women and the primary endpoint was response to the treatment as assessed by components of the Rome III criteria, while the number of bowel movements was not reported, this study confirms the beneficial effect of a natural mineral water rich in magnesium and sulphate on bowel function.

In the present study, a mineral water containing 13 g/L of dissolved mineral substances, among them magnesium sulphate and sodium sulphate was investigated. Magnesium sulphate exerts its osmotic effect by trapping water in the intestinal lumen, resulting in increased and softer faecal bulk, which presses on the intestinal wall provoking peristalsis [27, 28]. A study in healthy human subjects demonstrated that high doses of orally administered magnesium sulphate accelerate small intestinal transit time and modulate antroduodenal motility in the fasting but not in the postprandial state [11]. More recently, in vivo and in vitro studies showed that, along with the magnesium sulphate-induced change in the osmotic pressure, the expression of osmoregulatory genes is induced [29, 30]. In addition, the expression of Aquaporin 3, a gene involved in the regulation of faecal water content in the colon, was increased in the rat colon after administration of magnesium sulphate [30]. Another study in rats reported that the laxative effect of magnesium sulphate may involve the release of nitric oxide (NO) through the stimulation of NO synthase [31]. Sulphate is further known to increase faecal bulk and stool consistency, and water containing more than 1000 mg/L sulphate was linked to a self-reported laxative effect [32, 33]. Overall, the effect on stool frequency and consistency of the investigated mineral water is most likely related to its naturally high content of magnesium and sulphate.

Some limitations of the study should be noted. In addition to the effect of the mineral water on constipation, its influence on the entire gastrointestinal tract and the overall quality of life was evaluated. However, it might have been more appropriate to use constipation-specific questionnaires to assess the respective symptoms and quality of life rather than the more general tools applied in the study. Further, after 50 % of the originally planned number of subjects have been recruited, the lower-dose group was stopped based on the recommendation made by the IDMC. Yet, the sample size might have been too small to draw a firm conclusion for the 300-mL group. Further clinical trials with larger sample size might investigate an effect of the mineral-rich water. In addition, measuring other gut health-related parameters such as faecal bulk, transit time or a potential impact on microbiota would further elucidate the underlying mode of action.

In summary, the presented study in otherwise healthy subjects confirms the beneficial effect of drinking 500 mL natural mineral water daily on bowel function in subjects with functional constipation. In addition, the consumption of this specific mineral water was proven to be safe and tolerable. The observed results support the postulation that a mineral water naturally rich in magnesium sulphate and sodium sulphate may be considered as a first line of recommendation for subjects with less frequent bowel movements or harder stool in order to maintain a normal defecation. This in turn may help to reduce gastrointestinal discomfort and the development of diseases frequently associated with constipation. Finally, this might improve the health-related quality of life and subsequently reduce the economic burden on healthcare resources.
